# Utilisation of internet resources for continuing professional development: a cross-sectional survey of general practitioners in Scotland

**DOI:** 10.1186/s12909-016-0540-5

**Published:** 2016-01-21

**Authors:** Gordon MacWalter, John McKay, Paul Bowie

**Affiliations:** NHS Education for Scotland, 2 Central Quay, 89 Hydepark Street, Glasgow, G3 8BW UK; Institute of Health and Wellbeing, University of Glasgow, Glasgow, UK

**Keywords:** Online resources, Internet, e-learning, Continuing professional development, General practice

## Abstract

**Background:**

Participation in continuing professional development (CPD) is a professional and regulatory expectation of general practitioners (GPs). Traditionally, CPD activity was undertaken face-to-face in educational settings, but internet based formats have found increasing favour. The need for doctors to use the internet for service and educational purposes is growing, particularly in support of specialty training and appraisal. We aimed to determine how GPs in Scotland utilise online resources in support of their CPD. This involved identifying which resources are used and how frequently, along with their preferences as to how and why they access these resources.

**Methods:**

A cross sectional study was undertaken using an online questionnaire to survey general practitioners across Scotland. Data were subjected to descriptive analysis and differences in attitudinal responses between groups and Fischer's exact tests were calculated.

**Results:**

Three hundred and eighty-three GP responses were received, with the majority being female (*n* = 232, 60.6 %) and GP partners (*n* = 236, 61.6 %). The majority used the internet on three or more working days per week or more frequently (*n* = 361, 94.3 %) with the three most common reasons being to obtain information for a patient (*n* = 358, 93.5 %), answering a clinical question (*n* = 357, 93.2 %) and CPD purposes (*n* = 308, 80.4 %). Of 37 online resources used by respondents, the top five were SIGN Guidelines (*n* = 303, 79.3 %), BMJ Learning (*n* = 279, 73.0 %), NICE Guidelines (*n* = 255, 66.8 %), GP Notebook (*n* = 243, 63.6 %) and Google (*n* = 234, 61.3 %). Low use of social media such as Facebook (*n* = 11, 2.9 %) and Twitter (*n* = 11, 2.9 %) was reported for CPD. A majority agreed that 'reading information online' (95.0 %) and 'completing online learning modules' (87.4 %) were the most valued online activities. Slow internet connections (*n* = 240, 62.7 %), website access restrictions (*n* = 177, 46.2 %) and difficulties logging into online CPD resources (*n* = 163, 42.6 %) were reported barriers. Significant response differences (*P* < 0.05) were found between groups based on high volume online usage, gender and age.

**Conclusions:**

The majority of respondents had positive attitudes to using online resources for continuing professional development, and a preference for evidence-based and peer reviewed online resources. Information technology (IT) difficulties remain a barrier to effective utilisation. The findings have implications for future planning and design of online resources and IT infrastructure.

**Electronic supplementary material:**

The online version of this article (doi:10.1186/s12909-016-0540-5) contains supplementary material, which is available to authorized users.

## Background

Participation in continuing professional development (CPD) has long been part of the professional expectations of medical doctors, including general practitioners (GPs). CPD is defined by the Academy of Medical Royal Colleges (2009) as “*a continuing process that enables individual doctors to maintain and improve standards of medical practice through the development of knowledge, skills, attitudes and behaviour*” [1]. The CPD process is underpinned by the values of adult learning which should be self directed based on learning needs or development goals and be particularly relevant to frequently encountered tasks [2]. It is vitally important for medical professionals to continuously refresh, update and improve their clinical knowledge. This is problematic given that estimations suggest that by 2001 the volume of medical knowledge was doubling at least every 3.5 years, compared with every 50 years in the 1950s [3]. A more recent study has shown that global total scientific publication, including medical publication, continues to grow at a rate of 3 % annually from 1980 to 2012 [4].

The introduction of professional appraisal in 2004 required GPs to complete and record CPD activities as part of an annual educational review process. Medical revalidation, introduced in 2012 for all doctors in the United Kingdom, formalised this process with appraisal evidence supporting the periodic revalidation of doctors over a five-year cycle. The evidential appraisal process comprises six main elements: CPD, Quality improvement activity, Significant event analyses, Feedback from colleagues, Feedback from patients and Review of complaints and compliments [5].

Traditionally, CPD has been undertaken at medical conferences, professional meetings and included talks from experts, along with problem based small group learning (PBSGL) and reading professional printed journals. Advances in information technology (IT) have presented an opportunity for CPD learning to take place using online internet resources and in a less formally structured way.

Previous research has shown that internet based CPD formats can be equally effective as live face-to-face CPD sessions [6–8]. It has also been argued that less-structured learning such as using online resources should be eligible for CPD accreditation [9], as this involves complex high order cognitive processing (e.g. information searching and differentiating, and evaluating information sources). This is also reflected in the advice from the Academy of Medical Royal Colleges that “*self-accreditation of relevant activities and documented reflective practice should be allowed and encouraged*” for CPD purposes [1].

For the purposes of this study, the term “online resources” was used to describe all relevant World Wide Web based resources including, for example: clinical guidelines, patient resources, online simulated patient exercises, online learning modules, full-text journal articles and any other useful hyperlink or multimedia based websites. This approach was taken to distinguish from the concept of “e-Learning” which is difficult to define precisely [10] and often includes virtual learning environments (VLE) which are currently more commonly used at undergraduate level by higher education institutions, rather than for post-qualification CPD purposes [10].

General practitioners in the UK are required to submit a learning log of CPD at each appraisal, that should consist of 50 self-assessed credits, where each hour of learning activity accompanied with a reflective record is one learning credit [11].This differs from the use of certified CPD credits that have been utilised by medical professionals throughout the world, although internationally there is a shift to increased use of self-accreditation and reflection [12, 13]. The use of online resources is considered part of CPD for the purposes of this study; as such learning can be self-accredited by UK GPs provided there is an accompanying reflective record. Medical revalidation for licensure via the General Medical Council (GMC) is granted on a 5 year cycle, provided that GPs have participated in five annual appraisals covering an appropriate spread of their work [14].

The use of IT and the internet continues to rapidly increase in Scotland, with a survey in 2014 stating that 81 % of households in Scotland have access to the internet [15]. The use of IT in Scottish general practice was accelerated with the introduction of electronic record keeping in the mid 1980s [16]. Since then there has been a rise in the use of the internet within the medical profession as a whole with, for example, 79 % of GPs in Greater Glasgow being connected to the internet in 1999 via health board maintained IT [17]. From 2007 onwards, GP trainees have been required to keep an online electronic portfolio of evidence of learning as part of the basis for postgraduate license, making the use of the internet obligatory during training. Similarly, the Scottish online appraisal process necessitates the use of web based technologies, with participation also mandatory for all GPs in Scotland [18]. It can therefore be seen that use of IT and the internet has increased to the extent it is now inextricably linked with everyday general practice activities, including collating and submitting evidence of CPD.

The aim of this study was to determine how GPs in Scotland utilise online resources in support of their CPD by identifying which resources are used and how frequently, along with their preferences as to where and why they access these resources. It will also analyse the subgroups of the respondents to determine if there are differences in, or barriers to, the uptake of online resources. This is important as the results will help inform the future of CPD provision and delivery, as well as provide potential insights into possible training requirements for users in accessing online resources nationally. It may also serve to guide future developments in local health organisation IT provision and delivery. To our knowledge no recent research has been undertaken on this topic with this specific study population.

## Method

### Study design

A cross sectional online questionnaire survey was used.

### Development and testing of questionnaire

An online questionnaire was created by the authors using Questback software, comprising questions used in previous validated surveys on satisfaction with e-learning [19] and those developed by one of the investigators. The survey used a combination of true/false questions, tick all that apply and five-point Likert style responses. There were two sections for free text responses or comments. All responses were kept anonymous.

The draft survey was reviewed by a local clinical educator expert in e-learning and online resources. To inform face and content validity, it was then piloted in a regional educational office by GPs involved in CPD, appraisal and postgraduate training, as well as educational researchers and national practice manager and practice nursing educational leads. Revisions were made at this point to the wording, with clarifications and restructuring of the format of some questions. The final survey consisted of 21 items and had a completion time of approximately 10 mins. A copy of the questionnaire survey used is accessible in Additional file 1. 

### Setting, subjects and recruitment

GPs were recruited via electronic mailshot using a database from the national medical education organisation [20] for promoting CPD opportunities on a national basis to GPs, and member lists of three regional sessional and locum GP groups, as these individuals are often not included on such organisational lists. Sessional and Locum GPs are defined as GPs whom undertake work on a session by session basis, whom are not GP partners or salaried by one practice. Sessional GPs may work in GP practices as well as in hospital or educational roles, whereas Locum GPs would work in a variety of GP practices. After eliminating duplicates, a total of 3336 individual GPs were invited to complete the questionnaire via an email link sent in February 2015. The questionnaire was open for completion for a total of four weeks. A reminder email was sent after two weeks to those who had not already responded. Responses were motivated by goodwill, with no incentive for completion offered.

### Data analysis

The collected data was analysed and basic descriptive statistics generated including frequency counts and mean values related to individual question/statement responses. A 5-point Likert style scale was used alongside attitudinal statements (using a scale where 1 = strongly disagree and 5 = strongly agree) and the mean rating with standard deviation calculated. The proportion of responses rated as 4 or more was calculated as a proxy indicator of positive agreement with the statements posed. Fisher’s exact test was used to analyse comparative groups, with *P* < 0.05 deemed significant. This was performed using Microsoft Excel 2007 and IBM SPSS version 22.

### Ethical considerations

Under UK ‘Governance Arrangements for Research Ethics Committees,’ ethical research committee review is not required for service evaluation or research which, for example, seeks to elicit the views, experiences and knowledge of health care professionals on a given subject area [21]. Similarly ‘service evaluation’ that involves NHS staff recruited as research participants by virtue of their professional roles also does not require ethical review from an established NHS research ethics committee.

## Results

### Response rate and respondent characteristics

A total of 383 unique general practitioners responded, giving a response rate of 11.5 %. Respondents were more likely to be a member of the Royal College of General Practitioners (RCGP) (*n* = 248, 64.8 %), female (*n* = 232, 60.6 %) and to be a GP Partner (*n* = 236, 61.6 %). A full summary of respondent demographic details is outlined in Table 1.

**Table 1:** Demographic details of GP survey respondents

Characteristic	*n* = 383	%
Gender		
Female	232	60.6
Male	151	39.4
Age group in years		
25–34	53	13.8
35–44	107	27.9
45–54	127	33.2
55–64	91	23.8
>65	5	1.3
Role		
GP Partner	236	61.6
Locum GP	64	16.7
Salaried GP	47	12.3
Sessional GP	18	4.7
GP Retainer	15	3.9
GP Trainee	3	0.8
RCGP Status		
Member	248	64.8
Non-member	135	35.2
Hours of work		
Part-time	194	50.7
Full-time	189	49.3
Practice Location		
Inner city	78	20.4
Urban	150	39.2
Semi-rural	81	21.1
Remote and rural	36	9.4
Locum (varied)	38	9.9
GP involved in training of healthcare professionals		
Yes	156	40.7
No	227	59.3

### Internet usage and reasons for use

The majority of GPs reported that they used the internet for work related purposes every day (*n* = 273, 71.3 %), with 94.3 % (*n* = 361) indicating that they use the internet most days or every day. Respondents preferred to spend less than 60 mins at any one time using online resources (*n* = 338, 88.3 %), with the vast majority indicating that they would mostly use the resources when on their own (*n* = 372, 97.1 %). There was variation reported as to where respondents used online resources with 43.9 % (*n* = 168) indicating ‘mostly at home’, 33.2 % (*n* = 127) ‘either at home or at work’ and 23 % (*n* = 88) ‘mostly at work’.

The three most common reasons for using the internet were for obtaining information to give to a patient (*n* = 358, 93.5 %), answering a clinical question (*n* = 357, 93.2 %) and for self directed continuing professional development (*n* = 308, 80.4 %). A wide variety of online resources were used by respondents for work-related purposes, including CPD, which amounted to 37 unique sources in total (Table 2). The mean number of unique online resources used per respondent was seven, although the range amongst respondents was 0 to 17. Respondents reported using 37 unique online resources, the top five were SIGN Guidelines (*n* = 303, 79.3 %), BMJ Learning (*n* = 279, 73.0 %), NICE Guidelines (*n* = 255, 66.8 %), GP Notebook (*n* = 243, 63.6 %) and Google (*n* = 234, 61.3 %).

**Table 2:** Work related use of the internet reported by respondents and the online resources used

Use	*n* = 383	Percentage
Obtaining information to give to a patient	358	93.5 %
Answering a clinical question	357	93.2 %
Continuing professional development	308	80.4 %
Answering a non- clinical question	234	61.1 %
Literature searches	135	35.2 %
Other	91	23.8 %
None of the above reasons	1	0.3 %
Resource	*n* = 382	Percentage
SIGN Guidelines	303	79.3 %
BMJ Learning	279	73.0 %
NICE Guidelines	255	66.8 %
GP Notebook	243	63.6 %
Google	234	61.3 %
NICE Clinical Knowledge Summaries (CKS)	165	43.2 %
Healthcare Journals e.g. BMJ, Lancet, BJGP	159	41.6 %
RCGP Online Learning Environment	138	36.1 %
Doctors.net.uk	130	34.0 %
Wikipedia	116	30.4 %
NES Knowledge Network	81	21.2 %
Pulse Learning	75	19.6 %
GP Magazine / GP online and mycme.com	70	18.3 %
e-Learning for General Practice (e-GP)	67	17.5 %
LearnPro	63	16.5 %
e-Learning for Healthcare	56	14.7 %
GP Handbook	42	11.0 %
Other (not otherwise specified)	31	8.1 %
WebinarsForGPs.com	26	6.8 %
Another search engine other than Google	18	4.7 %
Patient.co.uk	13	3.4 %
Twitter	11	2.9 %
Facebook	11	2.9 %
Other specified (14 unique resources including: NB Hot Topics, Up To Date, Univadis, Dermnet NZ)	23	6.0 %

When using online resources, there was strong agreement that reading information online (95 % ≥ 4 valued, 94.5 % ≥ 4 frequently used) and utilising online learning modules (87.4 % ≥ 4 valued, 84.8 % ≥ 4 frequently used) were the most highly valued and commonly used. As social media can be seen a novel format for CPD, we included this in evaluation of the value and frequency of use. Respondents reported low value and use of both social media resources, Facebook and Twitter, for CPD purposes (Table 3).

**Table 3:** Type of online resources: perceived value and frequency of use of resource for CPD (*n* = 383)

	Value			Frequently use		
	Mean	Std. Deviation	% ≥ 4	Mean	Std. Deviation	% ≥ 4
Reading information online	4.40	.708	95.0	4.42	.677	94.5
Completing online learning modules	4.28	.807	87.4	4.16	.919	84.8
Simulated patient scenarios	3.55	.991	53.3	2.91	1.172	35.0
Watching online videos	3.52	.957	58.2	3.02	1.139	40.7
Reading a journal online	3.34	1.028	51.7	3.13	1.220	46.7
Participation in webinar	2.75	1.019	19.3	2.15	1.058	12.5
Online discussion board	2.56	.872	10.9	1.96	.942	6.8
Twitter	1.79	.973	5.2	1.52	.912	5.2
Facebook	1.72	.907	3.9	1.48	.824	4.4

(mean ratings, standard deviation and proportion of responses greater than 4 - based on Likert scale 1 = strongly disagree, 5 = strongly agree)

Barriers to use of online resources included technical issues such as connectivity, compatibility and problems with logging-in. Internet connection speed was deemed to be slow by 62.7 % (*n* = 240) of respondents, with a further 37.3 % (*n* = 143) reporting problems with internet connection other than speed. (Table 4)

**Table 4:** Reported difficulties encountered when using online CPD resources

	*n* = 383	Percentage
Slow internet connection	240	62.7 %
Needing additional software for browser	191	49.9 %
Access to website restricted by health board	177	46.2 %
Problems logging into online CPD resources	163	42.6 %
Internet connection problems other than speed	143	37.3 %
Incompatible browser	141	36.8 %
Problems with additional software downloaded	95	24.8 %
Problems logging into computer	51	13.3 %
Other (e.g. Incompatible with tablet computer, resource timing out, cannot access health board intranet from home, no audio output)	33	8.6 %

The vast majority of respondents stated that they enjoyed choosing resources relevant to their own learning (93 %), were comfortable using online resources (92.7 %), and use online resources opportunistically (90.1 %). Further attitudinal responses to online resources can be found in Table 5.

**Table 5:** GPs attitudes to online CPD resources

	Mean	Std. Deviation	% ≥ 4	% ≥ 4 % Comparison of high volume use versus low volume internet use for work purposes differences (*P*-value)	% ≥ 4 Comparison of groups aged 45 years and under against those over 45 years (*P*-value)	% ≥ 4 Comparison of male and female gender differences (*P*-value)	% ≥ 4 Comparison of partnership status differences (*P*-value)
I am comfortable using online resources	4.41	.746	92.7	<0.001^a^	0.002^b^	0.547	1.000
I like choosing online resources topics relevant to my learning	4.31	.689	93	0.059	0.004^b^	0.684	1.000
Sometimes I use online CPD resources opportunistically	4.20	.793	90.1	0.003^a^	0.056	0.729	0.863
Linking completed use of online resources into my CPD evidence would be useful to me	4.16	.860	84.1	0.063	0.257	0.198	0.317
I find using online CPD resources increases the accessibility of CPD topics	4.15	.823	84.9	<0.001^a^	0.388	0.384	0.771
I find using online resources saves me time	4.15	.904	81.5	<0.001^a^	0.005^b^	0.346	0.177
I usually find online resources are up to date	4.06	.672	83.8	0.375	0.888	0.571	0.255
I usually find online resources easy to use	4.01	.767	83.8	0.015^a^	0.016^b^	0.672	0.117
Using online resources saves me money	3.86	.973	68.7	0.002^a^	0.002^b^	<0.001^c^	0.013^d^
I usually find online resources enjoyable	3.80	.800	70.5	0.014^a^	0.174	0.022^c^	0.107
Using online resources helps with my work / life balance	3.79	.969	68.9	0.007^a^	0.010^b^	0.043^c^	0.650
Online resources allow me to plan my CPD work more efficiently	3.75	.893	66.8	0.010^a^	0.048^b^	0.096	0.316

(Based on Likert scale 1 = strongly disagree, 5 = strongly agree)

^a^Most days or every day use, ^b^age < 45 years old, ^c^Female, ^d^Not a GP partner

### Intergroup comparisons

Comparative analysis of GP respondent groupings demonstrated significant differences in GPs attitudes to online CPD resources (Table 5). For example, high volume internet users, identified by their use of the internet on three or more working days per week, showed significant tendencies to: be comfortable using online resources (*p* = <0.001), find using online resources increases accessibility of CPD topics (*p* = <0.001), saves time (*p* = <0.001) and money (*p* = 0.002) and finds online CPD resources enjoyable (*p* = 0.014) and easy to use (*p* = 0.015) compared to low volume users (use of the internet less than 3 days per working week). Respondents under the age of 45 were more likely than the older age group to be comfortable using online resources (*p* = 0.002), and agree that they helped with work/life balance (*p* = 0.01), were easy to use (*p* = 0.016) and allowed more efficient planning of CPD (*p* = 0.48). Female respondents were more likely to find using online resources helped with work/life balance (*p* = 0.043).

## Discussion

This study examined the frequency and type of use of online resources by a population of general practitioners based in Scotland. It showed that a variety of different styles of online resources are often utilised for CPD. The most commonly used resources included accessing clinical guidelines as well as those of leading professional bodies in the field. A non-medical search engine (Google) was also frequently used. In this study population, the flexibility, specificity of topic, opportunistic use and time were found to be important factors to using online resources.

The data show that 94 % of Scottish GP respondents reported that they use the internet on most days. This is higher than the 88 % reported in a study of GPs surveyed in Denmark in 2008 [22]. There is also in increase in *weekly* internet use (99 %) versus a population of German GPs (75 %), surveyed in 2009 [23]. However, both studies were over 5 years ago and it is likely that internet use has also increased in these study populations.

Online resources were most often used to obtain information for a patient or to answer specific clinical questions. This is perhaps unsurprising as it has been shown that over time, patient encounters have been increasingly generating more clinical questions that require additional information to be sourced quickly and in a readily accessible format by clinicians [24, 25].

The top five most frequently used resources included two clinical guideline resources - NICE guidelines [26] and SIGN guidance [27] – an international medical journal e-learning website (BMJ Learning) [28] and a point of care resource (GP Notebook) [29]. This suggests that GPs are choosing learning based on current best practice guidelines and preferring peer reviewed sources. The exception of this is GP Notebook, which has been described as being “unclear in peer review status” and “evidence base” in a recent review of point of care resources [30]. The use of guidelines as a preferred CPD resource may be a reflection of primary care in the UK being increasingly guideline driven, particularly with the introduction of the Quality Outcomes Framework, a pay for performance incentive that is largely guideline based [31].

In contrast, it was interesting to note the non-medical search engine Google also featured highly, with it ranked as the fifth most used resource. Previous research involving medical students use of Google suggested that the use of the search engine was down to perceived usefulness, rather than reliability of search results [32]. This raises some questions in relation to the information seeking behaviour in the GPs surveyed. This study did not assess how the search engine was used for CPD in detail. Previous research regarding doctors’ use of Google suggested that search engines were easier to use than research search resources such as PubMed and that the search engines would often link to PubMed in search results [33].

Despite widespread uptake of social media for private leisure use, it did not feature highly for use in CPD. The low uptake for social media platforms for professional use by doctors mirrors previous research which found comparatively low uptake of Facebook and Twitter compared with the general population. This might be explained by these platforms being deemed more social than professional, with potential adverse publicity and medico-legal complications with their use. As such it has been proposed that medical professionals might consider creating a separate account for more professional use of these platforms [34, 35].

It was evident that respondents were comfortable with the use of online resources. This is encouraging as a recent systematic review has shown that learning is more effective when the educational resources were chosen by the learners [36]. Although this is a key principle in adult learning (Knowles, 1980) in which learners want to choose what, how and where they learn [37], it is crucial that these resources are of high quality.

The frequency of technical issues encountered with using resources may well be an explanation for the varied locations of internet use reported. For example, it may be that users find internet browsing software and internet connections to be more stable at home and so find this to be a convenient location for learning. This concurs with a study amongst newly qualified doctors, that also showed a near equal distribution in location of use of an e-learning programme due to reasons of connectivity [38]. Previous research exploring GPs use of health informatics has called for increased training in IT skills for GPs, which may also help to reduce problems utilising online resources [16]. Whilst considering that investment in IT is a complex multi-organisational undertaking [16], stability of internet connection would appear to remain an issue.

It is unsurprising that higher users of the internet would find online resources useful to them. Younger GPs are more likely to have been users of the internet during previous aspects of their training or undergraduate studies, which may prepare them for continued use of online resources [23, 39]. The gender difference in attitudes may stem from differences in work pattern, with a higher proportion of female GPs working part-time, but also that female doctors have been shown to be early adopters of online CPD, with a diffusion of such resources within networks of female GPs a suggested reason [40].

### Study limitations

This study has highlighted the utilisation of online resources by 383 GPs for CPD purposes. The low response rate to the survey will introduce an aspect of non-responder bias. Furthermore, the CPD email distribution list used may well introduce an aspect of respondent bias of those with a particular interest in structured or formal CPD. Some addresses in the email shot were addressed to practice leads or managers, and therefore may not have been cascaded to GPs [41]. It could also be deduced that those that utilise online resources more often, will be more likely to respond to an online survey. For financial reasons it was not possible to introduce the control measure of a postal version of the questionnaire survey, to reduce any bias from higher volume users of the internet. A selection of online resource options was chosen as an aide memoire for respondents to indicate which resources they used. This will have introduced an aspect of bias in the selection, as more respondents will chose options available than if they had to record each resource used individually.

A greater proportion of females responded (60.6 %) than those on the GMC GP register (50.1 %). It is likely that a combination of the above factors contributed to the low response rate. Caution needs to be exercised with questionnaire surveys as data are self-reported and their veracity cannot be checked. The results are not generalisable to GPs in Scotland as a whole. Due to resource limitations, no further in depth statistical analysis was performed.

## Conclusions

The study shows that GPs are using online resources for CPD often and that these are well accepted and valued. However, attitudinal differences to online CPD resource utilisation between different GP groups and technical barriers to use were also evident. This study did not differentiate the preferences of online resources against face-to-face events or small group learning, an area that might be of interest to this population. However, the findings should be of interest to medical educators, health service organisations and policy makers involved in designing or supporting implementation of educational interventions across training, frontline service, CPD and regulatory environments.

## Additional file

Additional file 1:
**Questionnaire survey used in the study ‘Utilisation of internet resources for continuing professional development: a cross-sectional survey of general practitioners in Scotland’.** (PDF 21 kb)

## Abbreviations

CPD: continuing professional development
GMC: General Medical Council
GP: general practitioner
IT: information technology
PBSGL: problem based small group learning
RCGP: Royal College of General Practitioners
VLE: virtual learning environment
