# Supplementary material for: Utilisation of internet resources for continuing professional development: a cross-sectional survey of general practitioners in Scotland
Source: BMC Med Educ. 2016 Jan 21;16:24. doi: 10.1186/s12909-016-0540-5 (PMC4721189; doi:10.1186/s12909-016-0540-5)
Supplement: Additional file 1: — Questionnaire survey used in the study ‘Utilisation of internet resources for continuing professional development: a cross-sectional survey of general practitioners in Scotland’. (PDF 21 kb) [file 12909_2016_540_MOESM1_ESM.pdf]

Appendix 1 – Questionnaire used in study : Utilisation of internet resources for continuing professional development: a cross-sectional survey of general practitioners in Scotland.

Questions marked with an * are required to be answered

Welcome!

You have been invited to complete a short questionnaire about your use of resources for your continuing professional development (CPD)

The survey will take between 5 and 10 minutes to complete.

Your answers are important in helping shape the future of resources for CPD!

Many thanks.

Considering your internet usage

1) * In your workplace, how often do you use the internet for work related purposes?

- o every day
- o most days
- o once a week
- o less often than once a week
- o hardly ever

Consider your internet usage

2) * Do you use the internet for the following work related purposes? (select all that apply)

- o obtaining information to give to a patient
- o answering a clinical question
- o answering a nonclinical question
- o literature searches
- o continuing professional development
- o other
- o none of the above

Consider your sources of material from the following randomised list

3) Which of the following ONLINE resources have you used for your continuing professional development (CPD)? (select all that apply)

- o SIGN Guidelines
- o RCGP Online Learning Environment
- o Google
- o GP Magazine / GP online and mycme.com
- o NICE Guidelines
- o Doctors.net.uk
- o Healthcare Journals eg BMJ, Lancet, BJGP
- o Facebook
- o Twitter
- o NES Knowledge Network
- o eLearning for General Practice (eGP)
- o NICE Clinical Knowledge Summaries (CKS)
- o Webinars for GPs webinarsforgps.com
- o GP Notebook
- o another search engine other than Google
- o BMJ Learning
- o Wikipedia
- o eLearning for Healthcare
- o Pulse Learning
- o GP Handbook
- o LearnPro
- o Other (please specify) ___________________

Consider your environment

4) * Where are you when you use online resources for your CPD?

- o mostly at work
- o mostly at home
- o both home and work equally

Some doctors prefer to undertake CPD on their own, whilst others complete activities as a small team.

Consider the following statement...

5) * When using online CPD resources, I am

- o always on my own
- o mostly on my own
- o often with another person
- o always with another person

6) * On any one occasion, how long do you TEND to spend using online CPD resources?

- o less than 30 minutes
- o 30-60minutes
- o 61-90 minutes
- o 91-120 minutes
- o more than 120 minutes

7) * On any one occasion, how long would you LIKE to spend using online CPD resources?

- o less than 30 minutes
- o 30-60 minutes
- o 61-90 minutes
- o 91-120 minutes
- o more than 120 minutes

Information Technology problems

From time to time we all encounter problems with our IT systems.

8) * Have you encountered any of the following difficulties when trying to access online CPD resources?

- o Slow internet connection
- o Internet connection problems other than speed
- o Problems logging onto the computer
- o Problems logging into online CPD resources
- o Needing additional software downloaded (e.g. flash)
- o Problems with additional software downloaded (e.g. flash)
- o Access to website restricted by healthboard
- o Incompatible browser (e.g. Internet Explorer)
- o Other (see below)

9) Please describe any other difficulties you may have encountered when using online CPD resources.

Free text answer:____

Consider the format of online CPD resources

Please consider the following statement in relation to *your* continuing professional development (CPD) and indicate your level of agreement...

10) * I value the following resources for CPD

	Strongly disagree	disagree	Neither agree nor disagree	agree	Strongly agree
Reading information online					
Completing online learning modules					
Simulated patient scenarios					
Online discussion board					
Participation in webinar					
Watching online videos					
Reading a journal online					
Twitter					
Facebook					

Consider your internet usage

Please consider the following statement in relation to *your* continuing professional development (CPD) and indicate your level of agreement...

11) * I frequently use the following to support my CPD

	Strongly disagree	disagree	Neither agree nor disagree	agree	Strongly agree
Reading information online					
Completing online learning modules					
Simulated patient scenarios					
Online discussion board					
Participation in webinar					
Watching online videos					
Reading a journal online					
Twitter					
Facebook					

Consider your attitudes to online CPD resources

12) * Please indicate your level of agreement for the following statements...

	Strongly disagree	disagree	Neither agree nor disagree	agree	Strongly agree
I am comfortable using online resources					
I find using online resources saves me time					
Using online resources saves me money					
Sometimes I use online CPD resources opportunistically					
I find using online CPD resources increases the accessibility of CPD topics					
I like choosing online resources topics relevant to my learning					
I usually find online resources are up to date					
I usually find online resources easy to use					
I usually find online resources enjoyable					
Online resources allow me to plan my CPD work more efficiently					
Using online resources helps with my work / life balance					
Linking completed use of online resources into my CPD evidence would be useful to me					

Nearly there!

Now a little about you!

13) * Are you?

- o Male
- o Female

14) * Age? (years)

- o 16-24
- o 25-34
- o 35-44
- o 45-54
- o 55-64
- o 65+

15) * Years since graduation? _______years

16) Which of the following best describes your job role?

- o GP Partner
- o Salaried GP
- o Sessional GP
- o GP Retainer
- o Locum GP
- o GP Trainee

17) Do you consider yourself to be?

- o Fulltime
- o Part-time

18) * Are you a member of RCGP?

- o Yes
- o No

19) * Which of the following best describes your main practice location?

- o Inner city
- o Urban
- o Semi rural
- o Remote and rural
- o Locum

20) * Do you have any of the following roles? (tick all that apply)

- o Clinical Director
- o Training programme director
- o Educational supervisor
- o Undergraduate tutor
- o GP Appraiser
- o None of the above

21) Are there any aspects of using online CPD resources that we have not covered?

Free text: ____
